# The impact of RNA structure on coding sequence evolution in both bacteria and eukaryotes

**DOI:** 10.1186/1471-2148-14-87

**Published:** 2014-04-23

**Authors:** Wanjun Gu, Musheng Li, Yuming Xu, Ting Wang, Jae-Hong Ko, Tong Zhou

**Affiliations:** 1 Research Center for Learning Sciences, Southeast University, Nanjing, Jiangsu 210096, China; 2 School of Biological Sciences and Medical Engineering, Southeast University, Nanjing, Jiangsu 210096, China; 3 Department of Medicine, University of Arizona, Tucson, AZ 85721, USA; 4 Department of Physiology, College of Medicine, Chung-Ang University, Seoul 156-756, South Korea

**Keywords:** mRNA structure, Purifying selection, Synonymous mutation, Translation initiation, Codon usage bias, Gene expression

## Abstract

**Background:**

Many studies have found functional RNA secondary structures are selectively conserved among species. But, the effect of RNA structure selection on coding sequence evolution remains unknown. To address this problem, we systematically investigated the relationship between nucleotide conservation level and its structural sensitivity in four model organisms, *Escherichia coli*, yeast, fly, and mouse.

**Results:**

We define structurally sensitive sites as those with putative local structure-disruptive mutations. Using both the Mantel-Haenszel procedure and association test, we found structurally sensitive nucleotide sites evolved more slowly than non-sensitive sites in all four organisms. Furthermore, we observed that this association is more obvious in highly expressed genes and region near the start codon.

**Conclusion:**

We conclude that structurally sensitive sites in mRNA sequences normally have less nucleotide divergence in all species we analyzed. This study extends our understanding of the impact of RNA structure on coding sequence evolution, and is helpful to the development of a codon model with RNA structure information.

## Background

Messenger RNA (mRNA) encodes functional information with linear nucleotide sequences for amino acids in a cell. In addition to mRNA primary linear structure, base pairing of local nucleotides in mRNAs creates specific secondary structures, such as stems and loops. It has been reported that mRNA structures encode several regulatory information in different biological processes [1], including DNA transcription [2], pre-mRNA splicing [3], microRNA (miRNA) mediated gene regulation [4,5], gene translation [6-8], and cellular localization [9,10]. Experimental profiling of mRNA structure at genome scale both *in vitro*[6,11-13] and *in vivo*[14] has confirmed regulatory roles of mRNA secondary structure in various organisms. Given the essentiality of RNA structure in regulating gene expression, it is important to perform mRNA structure analysis from the perspective of evolution.

Current evolutionary studies on RNA structures can be largely classified into two groups. The first group focuses on the conservation level of RNA structures in a genome. The basic method is to compare RNA structures within species in a phylogenetic tree. A set of functional RNAs (fRNAs) with conserved structures have been identified in human [15-19], *Drosophila*[20], and yeast [21]. Although different algorithms were applied among these studies, the consensus conclusion of these studies is that RNA structures experienced widespread purifying selection in organisms. Unlike the studies we mentioned above, the main issue addressed in the second group of studies is the effect of RNA secondary structure change caused by a single-point mutation. Some SNPs in mRNA coding [22,23] and non-coding [24,25] regions can cause aberrant gene expression by affecting mRNA secondary structures. Also, a point mutation in or close to miRNA target sites would disrupt normal gene regulation by affecting local mRNA accessibility [5,26,27]. A more recent study investigated accumulated mutations in *Escherichia coli* (*E. coli*) genes over 40,000 generations of evolution, and found mutations that may disrupt mRNA secondary structure are selectively filtered out in the course of evolution [28].

As RNA secondary structure is conserved among species and the fitness of structurally disruptive mutations is low, it is reasonable to hypothesize that selection on RNA secondary structure should lead to less nucleotide sequence divergence in the genome. However, little is known on this topic so far. The only study, to our knowledge, was performed by Warden *et al.*[29]. They predicted fRNAs in coding region of yeast genes and found significant effects of RNA secondary structure on protein evolutionary rates [29]. Notably, RNA structures are selectively conserved in protein coding regions in many organisms, such as *Drosophila*[30], yeast [29] and human [16,17,31]. In a recent study, Smith *et al.*[16] proposed that the relative enrichment of conserved RNA structure was the highest in protein coding region than that in any other genomic region. To understand the effect of RNA structure conservation on coding sequence evolution, we systematically investigated the relationship between nucleotide conservation level and mRNA secondary structure in four model organisms, including one prokaryote and three eukaryotes, *E. coli*, *Saccharomyces cerevisiae* (*S. cerevisiae*), *Drosophila melanogaster* (*D. melanogaster*), and *Mus musculus* (*M. musculus*). We define structurally sensitive sites in mRNA as those with putative local structure-disruptive mutations. We first assess whether structurally sensitive nucleotide sites are more conserved than non-sensitive sites. Next, we compare the above effect between genes with different expression level and codon usage bias, respectively. To further elucidate sequence constraint in different local regions along mRNA, we analyze the effect in translation initiation and elongation regions. Our analyses present a deep view of structure-associated nucleotide divergence in coding region. This study extends our understanding on the evolutionary process of coding sequences and helps develop a better model for coding sequence evolution.

## Results

### Structurally sensitive sites in mRNA are more evolutionarily conserved

We first assessed whether the mRNA nucleotide sites with putative structurally disruptive mutations are more evolutionarily conserved. According to the table of genetic code, most substitutions at the first codon position and all the substitutions at the second codon position are nonsynonymous. The conservation level of nucleotide at the first and second codon position is inevitably governed by strong purifying selection against amino acid replacement. To avoid the confounding factors caused by the selection on nonsynonymous sites, only the 4-fold degenerate sites in coding sequences were investigated in this study, which means we focused on the synonymous sites without any nonsynonymous mutational opportunity.

We evaluated the conservation level of each 4-fold degenerate site by weighted entropy (*E*_*w*_), which was calculated by multiple sequence alignment of widely diverged orthologs (see Methods for details). Lower *E*_*w*_ means higher conservation level and *E*_*w*_ = 0 means the no variation in nucleotide type in the alignment column. Here, we considered sites with *E*_*w*_ < 0.5 as conserved. For comparison, we also computed phyloP score [32] for each nucleotide site, which is a conservation score based on a model of neutral evolution (see Methods for details). Higher phyloP score means higher conservation level. We found a very strong negative correlation between *E*_*w*_ and phyloP score, with the mean of Pearson correlation coefficient < −0.85 across all the genes in each species (Additional file 1: Figure S1).

We used RNAsnp [33] program to assess the effect of single-point mutation on local mRNA secondary structure. RNAsnp helps screen the putative structure-disruptive mutations in RNA sequences by estimating the structural changes of all three possible substitutions at each nucleotide site. The structural distance (*d*_*max*_) between wild-type and mutant sequences was calculated from base pairing probability matrices [33]. We measured the structural sensitivity for a 4-fold degenerate site by the mean *d*_*max*_ for all 3 possible mutations at this site, assessing the likelihood that a mutation at this site is structurally disruptive. We considered a nucleotide site as structurally sensitive if the structural sensitivity was larger than 0.1.

**Figure 1:**
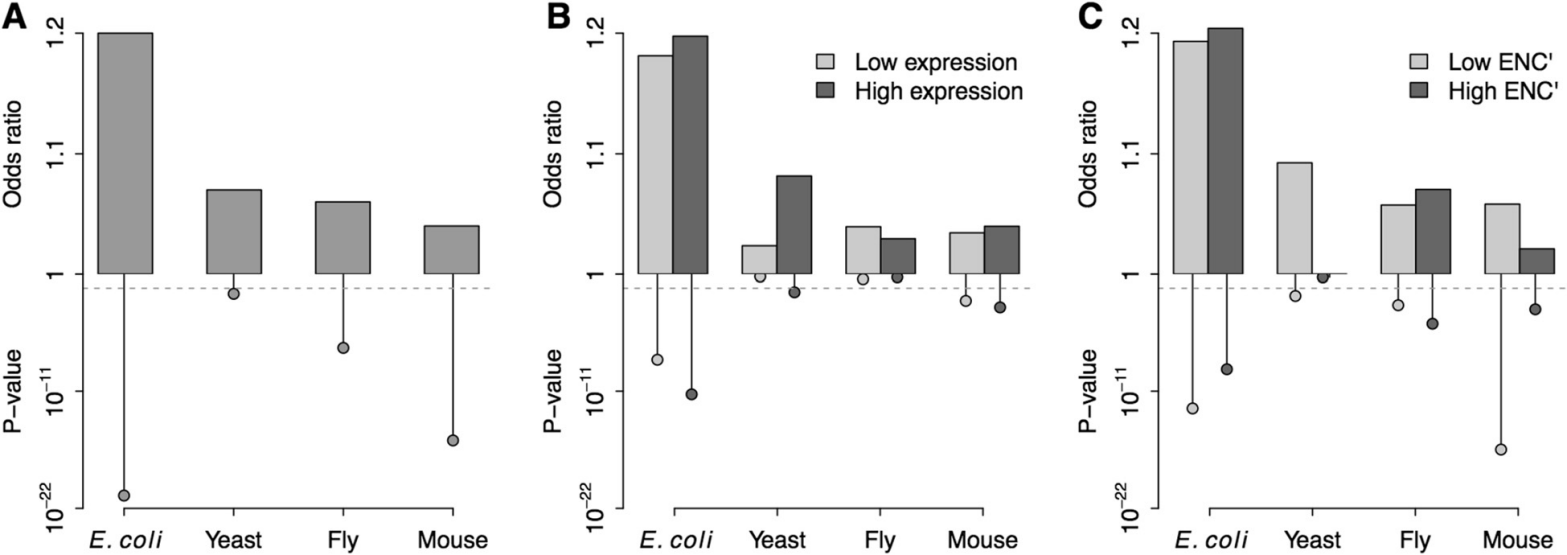
**Odds ratios and significance levels generated by Mantel-Haenszel procedure. A)** Comparison between species; **B)** Comparison between the 50% highest and lowest expressed genes; and **C)** Comparison between the genes with the top and bottom 50% *ENC’* level. The dashed line denotes the significance level of α = 0.05.

For each gene, we constructed a 2 × 2 contingency table by categorizing each 4-fold degenerated sites as structurally sensitive/non-sensitive and as evolutionarily conserved/non-conserved (Additional file 2: Table S1 for an example). We employed Mantel-Haenszel procedure [34,35] to determine whether structurally sensitive nucleotide sites are more evolutionarily conserved. A joint odds ratio (*OR*_*MH*_) was computed for each species by combining the odds ratios of each individual contingency table. *OR*_*MH*_ greater than 1.0 signifies that structurally sensitive nucleotide sites tend to be more conserved than non-sensitive sites. Mantel-Haenszel procedure reveals that, in all organisms, the *OR*_*MH*_ was significantly larger than 1.0 (*OR*_*MH*_ =1.20, *P* = 1.6 × 10^−21^ for *E. coli*; *OR*_*MH*_ =1.07, *P* = 1.5 × 10^−2^ for yeast; *OR*_*MH*_ =1.06, *P* = 1.2 × 10^−7^ for fly; and *OR*_*MH*_ =1.04, *P* = 2.3 × 10^−16^ for mouse) (Figure 1A). The 95% confidence interval of *OR*_*MH*_ is (1.15, 1.24) for *E. coli*, (1.01, 1.12) for yeast, (1.04, 1.09) for fly, and (1.03, 1.05) for mouse. These results were not strongly dependent on the cutoff choice for weighted entropy and structural sensitivity (Additional file 2: Table S1). A different choice of cutoffs only led to slightly different results.

To investigate the reason why *E. coli* shows the most significant signal while yeast shows the least significant signal, we counted the number of structurally sensitive sites for each gene within each species. *E. coli* shows the highest fraction of sensitive sites, while the proportion in yeast was significantly lower than that in the other species (*P* < 10^−8^ by t-test; see also Additional file 3: Figure S2). Since the power of Mantel-Haenszel test is affected by the number of sensitive sites, the lowest number of sensitive sites in yeast may partly explain the least significant signal in this species.

To determine whether the conservation level at structurally sensitive sites was affected by expression level, we calculated the *OR*_*MH*_ separately for the genes with the highest 50% and the lowest 50% expression level. In all the species except fly, the *OR*_*MH*_ for the highest-expressed genes tended to be higher than that for the genes with the lowest expression level (Figure 1B). The corresponding *P*-values were also more significant in highly expressed genes in all species except fly (Figure 1B).

We also tested whether gene codon bias could affect the conservation level at structurally sensitive sites. Effective number of codons (*ENC*) is usually used to measure gene codon usage bias [36]. Here, we used an improved version of *ENC*, *ENC’*, which takes background nucleotide composition into account [37,38]. Lower *ENC’* values indicate stronger codon bias. By comparing the bottom 50% of genes with the lowest *ENC’* to the top 50% of genes with the highest *ENC’*, we found that, in all the species with the exception of fly, the *P*-values for the genes with stronger codon bias tended to be more significant than those for the genes with the lowest codon bias (Figure 1C).

In addition, we repeated the above analyses using phyloP score as the measure of nucleotide conservation level (Additional file 4: Figure S3). We considered sites with phyloP score > 0 as conserved. Side-by-side comparison between Figure 1 and Additional file 4: Figure S3 indicated that the results generated based on phyloP score mirrored what we found when using weighed entropy as the measure of conservation level.

### Stronger association between conservation level and structural sensitivity at translation initiation region

**Figure 2:**
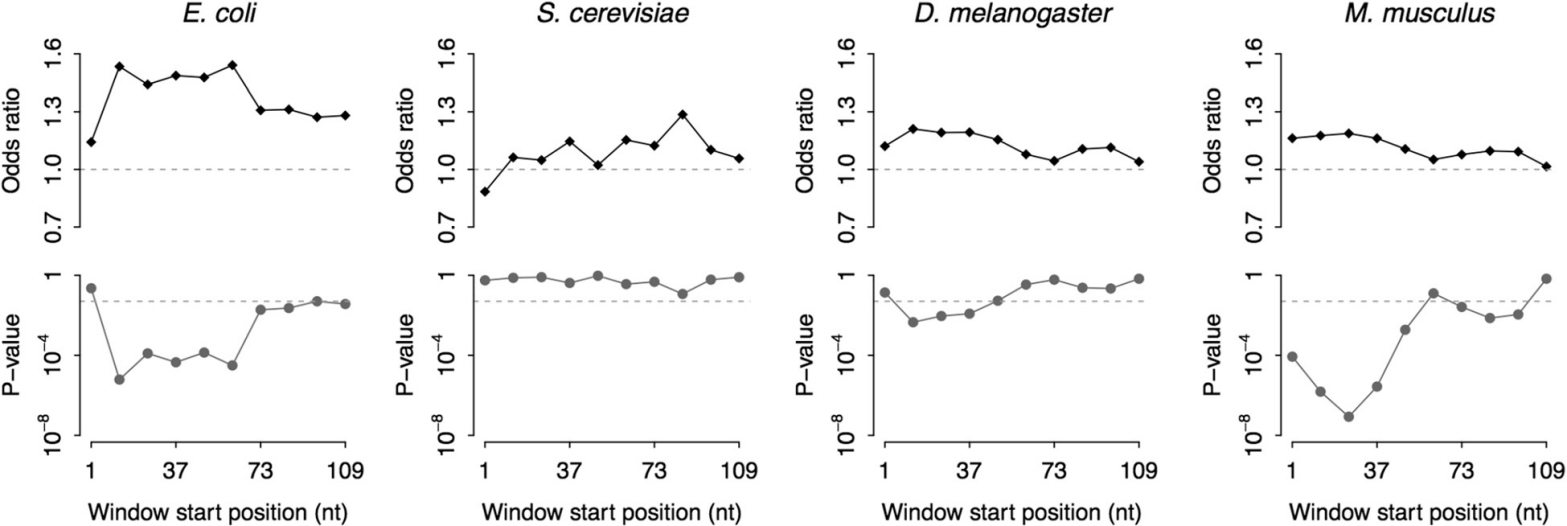
**The odds ratio and significance level of the 5′ sliding windows.** We conducted Mantel-Haenszel test along the mRNA sequence using a sliding window of 36 nucleotides (nt) in length, moving from the start codon to the 109th downstream nucleotide in steps of 12 nt (for a total of 10 windows). The dashed lines in the lower panels denote the significance level of α = 0.05.

A general feature of depletion of strong secondary structures has been found in mRNA translation initiation region in viruses [39], prokaryotes [40], and eukaryotes [7,41]. To further elucidate regional constraints along mRNA sequence, we checked the relationship between nucleotide conservation level and structural sensitivity at the 5′ end of the coding region in each species. Mantel-Haenszel procedure was conducted along the mRNA sequence using a sliding window of 36 nucleotides in length, moving from the start codon to the 109th downstream nucleotide in step of 12 nucleotides (for a total 10 windows). Figure 2 shows the odds ratio and corresponding *P*-value of each window. In all species except yeast, we observed an increased odds ratio and significance level for the windows close to the translation start site (except the first window), comparing with the downstream windows (from the seventh window to the tenth window).

To investigate whether window size affected our results, we redid our analysis for the four species using sliding windows of 45 nucleotides. Results for the alternate window size were compatible to those obtained with a window size of 36 nucleotides (Additional file 5: Figure S4).

To address why there is an exceptional pattern in yeast, we checked the composition of structurally sensitive sites for each window. Similar to the results mentioned in the previous subsection, the fraction of sensitive sites of the 5′ sliding windows was lower in yeast compared with the other species (Additional file 6: Figure S5). The lowest number of sensitive sites in yeast may interpret the least significant signal in this species. Also, we observed a trend that there are more structurally sensitive sites in the 5′ end windows, comparing with the downstream windows from the same species (Additional file 6: Figure S5).

### Weighted entropy correlates negatively with structural sensitivity

All above analyses were based on categorized data, such as a classification of all nucleotide sites into conserved/non-conserved or structurally sensitive/non-sensitive. Weighted entropy and structural sensitivity are continuous quantities. Lower weighted entropy denotes higher conservation level while higher structural sensitivity indicates more severe structural constraints. Therefore, if forcing both variables into dichotomous categories, we may lose statistical power.

To make use of the continuous values of structural sensitivity and weighted entropy for each nucleotide site, we calculated the Pearson correlation coefficient between structural sensitivity and weighted entropy of the 4-fold degenerate sites in each gene. As test statistic, we used the mean of all these correlation coefficients. We calculated the sampling distribution of this statistic by randomly permuting weighted entropy of 4-fold degenerate sites with identical nucleotide within each gene. Since we expected weighted entropy to decrease with structural sensitivity, we calculated one-tailed *P*-values for the left tail of the sampling distribution of the mean correlation coefficient. Our alternative hypothesis was that the mean correlation coefficient should be more negative than expected by chance if structural sensitive sites are more evolutionarily conserved.

**Figure 3:**
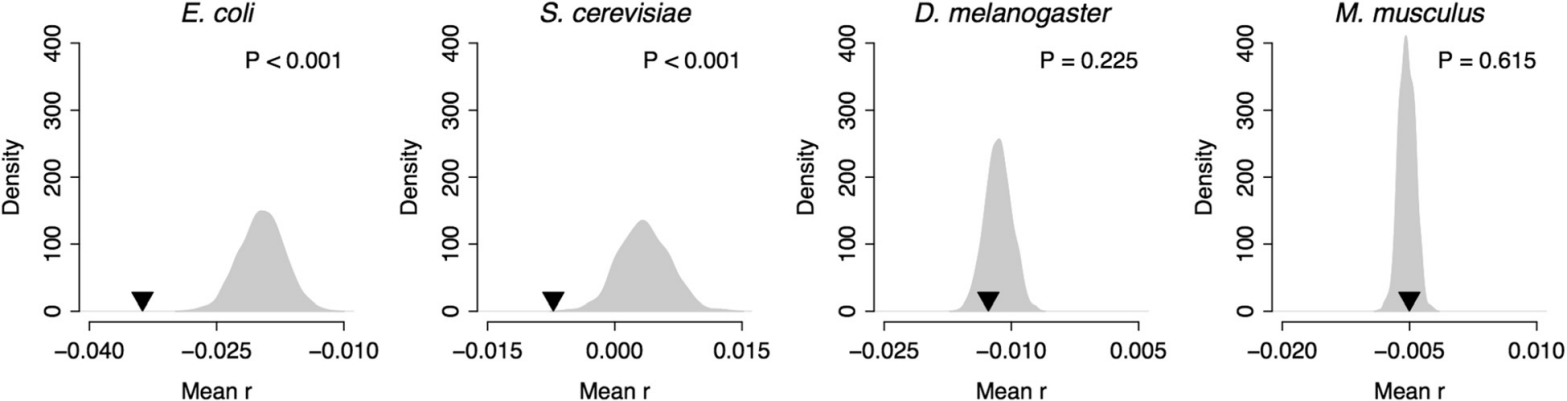
**Test for association between weighted entropy and structural sensitivity.** The black triangles indicate the mean Pearson correlation coefficient between these two quantities over all genes. The light grey areas show the sampling distribution of the same quantity under the null hypothesis of no association.

We found that, for *E. coli* and yeast, we could reject the null hypothesis of no significant association between weighted entropy and structural sensitivity (*P* < 0.001 for both species) (Figure 3). However, there is no significant association between the two quantities for fly and mouse (*P* = 0.225 for fly and *P* = 0.615 for mouse) (Figure 3).

**Figure 4:**
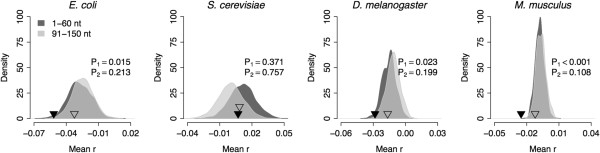
**Association between weighted entropy and structural sensitivity for the 5′ end of the coding region.** The solid black triangles indicate the mean correlation coefficient between these two quantities for the 5′ coding region from the first to the 60th nucleotide. The empty triangles indicate the mean correlation coefficient for the 5′ coding region from the 91st to the 150th nucleotide. The dark and light grey areas show the sampling distribution of the same quantity under the null hypothesis of no association for both regions, respectively. *P*_*1*_: the left-tailed *P*-values for the region between the 1st and the 60th nucleotide; *P*_*2*_: the left-tailed *P*-values for the region between the 91st and the 150th nucleotide.

To test whether there is difference between translation initiation and elongation regions, we carried out the same continuous test for the regions between the 1st and 60th nucleotides (initiation) and between the 91st and 150th nucleotides (elongation), respectively. Interestingly, we could reject the null hypothesis of no significant association between weighted entropy and structural sensitivity at translation initiation region for all the species with the exception of yeast (*P* = 0.015 for *E. coli*, *P* = 0.371 for yeast, *P* = 0.023 for fly, and *P* < 0.001 for mouse) (Figure 4). However, there is no significant association between the two quantities at translation elongation region for all the species (*P* = 0.213 for *E. coli*, *P* = 0.757 for yeast, *P* = 0.199 for fly, and *P* = 0.108 for mouse) (Figure 4).

## Discussion

We examined the relationship between the conservation level of 4-fold degenerate sites and the corresponding structural sensitivity in the mRNAs in four model organisms. Using both categorized and continuous analyses, we found that the conservation level is increased for the nucleotide sites with putative structurally disruptive single-point mutations. In *E. coli*, yeast, and mouse, the association is stronger in highly expressed genes than in genes with low expression level. Also, we found that the association is stronger at 5′ translation initiation region comparing with downstream elongation sequence. All these findings suggest that structurally important sites tend to experience stronger purifying selection at the nucleotide level from prokaryotes to eukaryotes.

In Mantel-Haenszel procedure, we used a cutoff to categorize the 4-fold codon sites into two groups: structurally sensitive vs. non-sensitive. It was suggested by the authors of RNAsnp that roughly 8-9% single-point mutations in RNA are structurally disruptive [33]. Because there are three possible mutations at a nucleotide site, we can reasonably expect that, on average, there are less than ~25% nucleotide sites in mRNA with potentially disruptive mutations. In our study, the proportion of structurally sensitive site in most mRNAs was lower than 25% (Additional file 3: Figure S2), which suggests that we chose a reasonable cutoff for structural sensitivity. In addition, less stringent cutoffs for structural sensitivity only slightly changed the results (Additional file 2: Table S1), which suggest that our results were independent of the cutoff choice.

Protein functional and structural constraints govern the evolution at nonsynonymous sites in coding sequences. Therefore, we didn’t take the first and second codon positions into account. We only focused on the 4-fold degenerate sites. However, it is important to note that 4-fold degenerate sites are not essentially free of selective constraints. Several mechanisms have been reported, which cause selective pressure on synonymous sites, such as selection for accurate and fast translation [42-47], selection for RNA global stability [48], selection for miRNA binding [5], selection for splicing efficiency [49,50], and selection for protein co-translational folding [51-53]. All these factors may weaken or bias the association between local structural sensitivity and site conservation level. In the resampling test, we kept the amino acid sequence, codon usage bias, and nucleotide composition for each gene, which helps avoid some of the confounding selective factors acting on synonymous sites.

There is a general observation that evolutionary constraints appear to increase with gene expression level [45,54-56]. Our results also indicate that highly expressed genes seem to exhibit a stronger association between conservation level and structural importance than genes with low expression level. The exception of fly may be due to the fact that the fly dataset with available expression information was extremely shrunk. Given the fact that codon bias somewhat reflects gene expression level [57-59], the effect of *ENC’* largely mirrors our findings on gene expression.

We found a stronger association between nucleotide conservation level and structural sensitivity at 5′ translation initiation region comparing with downstream elongation region, which is likely due to the enhanced importance of mRNA secondary structure for translation initiation. Several recent studies have demonstrated various structure-related regulatory mechanisms in mRNA translational process [7,31,60-64]. Especially, a universal selection on reduced RNA stability at translation initiation region has been reported from prokaryotes to eukaryotes by *in silico* studies [7]. Experimental studies also confirmed the key role of RNA structure near the start codon for translation initiation [61,62,65]. Some regulatory structures are also observed near the translation initiation region, such as internal ribosomal entry sites (IRES) in some eukaryotic genes [66] and PKR activating structure in inflammation-related genes [67]. Unlike translation initiation, codon usage and corresponding tRNA abundance, rather than RNA secondary structure, are the more important factors that regulates translation elongation and the final output of gene expression [61]. Therefore, it’s not surprising that the association of RNA structural sensitivity with nucleotide conservation is stronger at translation initiation region.

Our results suggest a universal trend of increased nucleotide conservation at structurally sensitive nucleotide sites. But, the statistical significance is weakest in yeast, which may be due to the lower fraction of sensitive sites in yeast. Both global and sliding window analyses indicate that the proportion of structurally sensitive sites was significantly reduced in yeast compared with the other species (Additional file 3: Figure S2 and Additional file 6: Figure S5). The relatively low number of sensitive sites could decrease the statistical power of our analysis, which may partly explain why the signal in yeast is kind of blurred.

Although this study is not the first to provide the evidence that protein-coding sequences are under evolutionary selection in keeping functional RNA secondary structure, we found a relatively strong and pervasive signal that structurally important sites tend to be more evolutionarily conserved from prokaryotes to eukaryotes, which is stronger for highly expressed genes and for translation initiation region.

## Conclusions

Our results highlight the importance of local RNA secondary structure in coding sequence evolution, and suggest that mRNA sequences are experiencing purifying selection in keeping functional RNA secondary structures. The inclusion of local RNA secondary structure information in a codon model should be beneficial for the detection of purifying/positive selection in coding sequences.

## Methods

### Genomic data

We obtained genomic sequences from the following sources: the Comprehensive Microbial Resource (http://cmr.tigr.org/) for *E. coli*, the *Saccharomyces* Genome Database (ftp://genome-ftp.stanford.edu/) for *S. cerevisiae*, the Eisen Lab (http://rana.lbl.gov/drosophila/) for *D. melanogaster*, and Ensembl (http://www.ensembl.org/) for *M. musculus*.

For *E. coli*, we obtained orthologs in *Shigella sonnei*, *Shigella flexneri*, *Shigella boydii*, *Shigella dysenteriae*, *Klebsiella pneumoniae*, *Salmonella typhimurium*, *Salmonella enterica*, *Photorhabdus luminescens*, and *Sodalis glossinidius* from TIGR’s Comprehensive Microbial Resource (http://cmr.tigr.org/). For *S. cerevisiae*, we obtained orthologs in *Saccharomyces paradoxus*, *Saccharomyces mikatae*, *Saccharomyces bayanus*, *Saccharomyces kudriavzevii*, *Saccharomyces castellii*, and *Saccharomyces kluyveri* from the *Saccharomyces* Genome Database (ftp://genome-ftp.stanford.edu/). For *D. melanogaster*, we obtained orthologs in *Drosophila simulans*, *Drosophila sechellia*, *Drosophila yakuba*, *Drosophila erecta*, *Drosophila ananassae*, *Drosophila pseudoobscura*, *Drosophila persimilis*, *Drosophila willistoni*, *Drosophila mojavensis*, *Drosophila virilis*, and *Drosophila grimshawi* from the Drosophila 12-genome project AAAWiki at http://rana.lbl.gov/drosophila/. For mouse, we obtained orthologs in human, chimp, macaque, rat, cow, dog, and horse from Biomart through the Ensembl Homology track (http://www.ensembl.org/). We built multiple alignments of orthologous sequences based on the peptide sequences with MUSCLE [68]. We excluded from our data set those ortholog pairs for which less than 80% of either sequence could be aligned to the other sequence. We only saved the alignments in which each species has its corresponding ortholog. This step yielded 1,156, 1,164, 3,047, and 6,324 alignments in *E. coli*, yeast, fly, and mouse, respectively.

### Nucleotide site conservation level

For each species group, the evolutionary phylogenetic tree was inferred by RAxML [69] using concatenated amino acid sequence (Additional file 7: Figure S6). Based on the topology and branch lengths of the tree, weights were be calculated by Branch Manager [70] for each species in the alignment that control for phylogenetic relationship among the orthologous sequences. Then the conservation level of a particular nucleotide site in the alignment can be expressed as weighted entropy (*E*_*w*_):

$$E_{w}=-\sum_{i\in N}p_{i}\log_{2}p_{i}$$

Here, *N* is the set of unique nucleotides in the column and *p*_*i*_ is the weighted fraction of sequences carrying a particular nucleotide *i*. Lower *E*_*w*_ means higher conservation level and *E*_*w*_ = 0 means the no variation in nucleotide type in the column. We considered sites with *E*_*w*_ < 0.5 as conserved.

We also applied phyloP program [32] to compute conservation score for each nucleotide site. The conservation *P*-values were computed using the likelihood ratio test (LRT) method with “--wig-scores” option. The phylogenetic model was produced by the phyloFit program [71] using “REV” nucleotide substitution model. The site specific conservation score was computed as “-log(*P*)”. Higher phyloP score means higher conservation level.

### Expression data

We used previously published expression data for each species: for *E. coli*, we obtained gene expression levels measured in mRNAs per cell from [72]; for *S. cerevisiae*, we used expression data from [73]; for *D. melanogaster*, we used as expression level the geometric mean of expression data from different tissues obtained by [74]; and for *M. musculus*, we measured expression level as the breadth of expression among different tissues [75].

### Mutation-induced mRNA structural change

We used the RNAsnp to estimate local mRNA secondary structural changes induced by mutations. This program focuses on the local regions of maximal structural change between mutant and wild-type [33]. We applied “Mode 3″ in RNAsnp with default settings to screen putative structure-disruptive mutations in mRNA sequences. The mutation effects were quantified by maximum structural distance (*d*_*max*_). We measured the structural sensitivity for a nucleotide site by the mean *d*_*max*_ for all 3 possible mutations at this site. We considered a nucleotide site as structurally sensitive if the structural sensitivity was larger than 0.1.

### Statistical analysis

To avoid the confounding factors, such as amino acid composition and strong purifying selection on nonsynonymous sites, we only focused on the synonymous sites without any nonsynonymous mutational opportunity. This means only the 4-fold degenerate sites were taken into account in this study (Additional file 8: Table S2). The percentage of 4-fold degenerate sites among the third codon positions of each gene varies from roughly 20% to 70% (Additional file 9: Figure S7). In total, 200,786, 160,079, 657,900, and 1,598,517 4-fold degenerate sites were included for *E. coli*, yeast, fly, and mouse, respectively.

We used two different statistical methods to test the association between site conservation level and structural sensitivity. The first method was to use discrete variables. We stratified the weighed entropy and structural sensitivity, and constructed a separate 2 × 2 contingency table for each gene (Table 1). We then combined the tables for all genes into an overall analysis, using the Mantel-Haenszel procedure [34,35]. *OR*_*MH*_ was computed by combining the odds ratios of each individual contingency table. As can be seen in Table 2, for one such contingency table *i*, the counts of the conserved (*a*_*i*_ or *c*_*i*_) and non-conserved (*b*_*i*_ or *d*_*i*_) sites were recorded. *s*_*i*_ stands for the total count of the *i*^th^ contingency table. Using the count from Table 2, the *OR*_*MH*_ is given by:

$$OR_{\mathrm{MH}}=\sum_{i}\frac{a_{i}d_{i}}{S_{i}}/\sum_{i}\frac{b_{i}c_{i}}{S_{i}}$$

**Table 1:** **2 × 2 contingency table for one particular gene in *E. coli***

	**Conserved sites**	**Non-conserved sites**
Structurally sensitive	3	10
Structurally non-sensitive	4	57

Note - The odds ratio of conservation pattern between structurally disruptive and non-disruptive sites is (3/10)/(4/57) = 4.28. Because there is one contingency table per gene, we applied the Mantel-Haenszel test to compute the joint odds ratio across all genes.

**Table 2:** **Counts for the *i***^**th **^**2 × 2 contingency table**

	**Conserved sites**	**Non-conserved sites**	**Total**
Structurally sensitive	*a*_*i*_	*b*_*i*_	*m*_*1i*_
Structurally non-sensitive	*c*_*i*_	*d*_*i*_	*m*_*2i*_
Total	*n*_*1i*_	*n*_*2i*_	*s*_*i*_

The null hypothesis in this analysis assumes that the conservation status of 4-fold degenerate sites (e.g. conserved or non-conserved) is independent of the corresponding structural status (e.g. structurally sensitive or non-sensitive) in any given stratum. The Mantel-Haenszel procedure was conducted by “mantelhaen.test” function in R plotform with the options of continuity correction and “two.sided” alternative hypothesis.

The second method was to calculate the Pearson correlation coefficient between the two continuous variables (weighed entropy and structural sensitivity) for each gene. As test statistic, we used the mean of the correlation coefficients over all genes. We calculated the sampling distribution by randomly reshuffling, separately for each gene, weighted entropy among 4-fold degenerate sites with identical nucleotide and recalculating all correlation coefficients. We generated 1,000 resampled sequences for each gene. All the statistical analyses were conducted using the R platform (version 2.15.1).

## Availability of supporting data

The data sets supporting the results of this article are available in TreeBASE, http://purl.org/phylo/treebase/phylows/study/TB2:S15642.

## Competing interests

The authors declare that they have no competing interest.

## Authors’ contributions

WG conceived the study, performed the analyses, analyzed the data, created the figures and wrote the paper. ML, YX and TW performed the analysis, analyzed the data. JK and TZ initiated the study, analyzed the data, created the figures and wrote the paper. All authors read and approved the final manuscript.

## Supplementary Material

Additional file 1: Figure S1 Distribution of Pearson correlation coefficient between phyloP score and weighted entropy. Pearson correlation test was conducted for each gene. The dash line indicates the mean of Pearson correlation coefficient. Click here for file

Additional file 2: Table S1 Odds ratio of conservation pattern between structurally disruptive and non-disruptive sites using different cutoffs. Click here for file

Additional file 3: Figure S2 Fraction of structurally sensitive sites in each species. We considered a nucleotide site as structurally sensitive if its structural sensitivity is larger than 0.1. Click here for file

Additional file 4: Figure S3 Odds ratios and significance levels generated by Mantel-Haenszel procedure. We used phyloP conservation score as the measure of nucleotide conservation level. We considered sites with phyloP score > 0 as conserved. A) Comparison between species; B) Comparison between the 50% highest and lowest expressed genes; and C) Comparison between the genes with the top and bottom 50% *ENC’* level. The dashed line denotes the significance level of α = 0.05. Click here for file

Additional file 5: Figure S4 The odds ratio and significance level of the 5′ sliding windows. We conducted Mantel-Haenszel test along the mRNA sequence using a sliding window of 45 nucleotides (nt) in length, moving from the start codon to the 121st downstream nucleotide in steps of 15 nt (for a total of 9 windows). The dashed lines in the lower panels denote the significance level of α = 0.05. Click here for file

Additional file 6: Figure S5 Fraction of structurally sensitive sites of the 5′ sliding windows. We calculated the fraction of sensitive sites along the mRNA sequence using a sliding window of 36 nucleotides (nt) in length, moving from the start codon to the 109th downstream nucleotide in steps of 12 nt (for a total of 10 windows). Click here for file

Additional file 7: Figure S6 Phylogenetic tree inferred by RAxML. Each phylogeny was estimated using the PROTGAMMABLOSUM62 model in RAxML. Click here for file

Additional file 8: Table S2 Codons with 4-fold degenerate sites. Click here for file

Additional file 9: Figure S7 Distribution of the proportion of 4-fold degenerate sites among the third codon positions in each gene. Click here for file
