# Supplementary figures and images for: The impact of RNA structure on coding sequence evolution in both bacteria and eukaryotes
Source: BMC Evol Biol. 2014 Apr 23;14:87. doi: 10.1186/1471-2148-14-87 (PMC4021280; doi:10.1186/1471-2148-14-87)

*E. coli*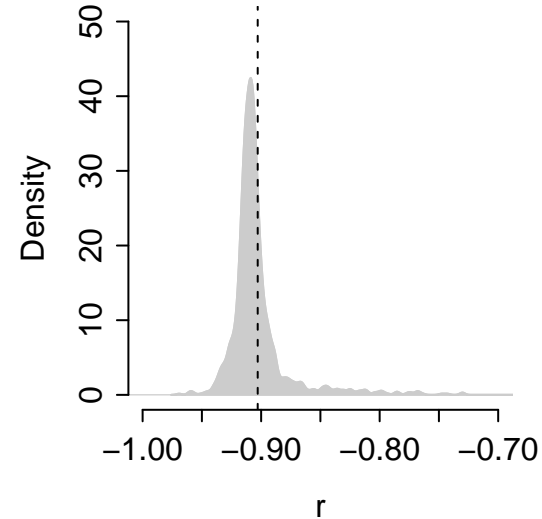*S. cerevisiae*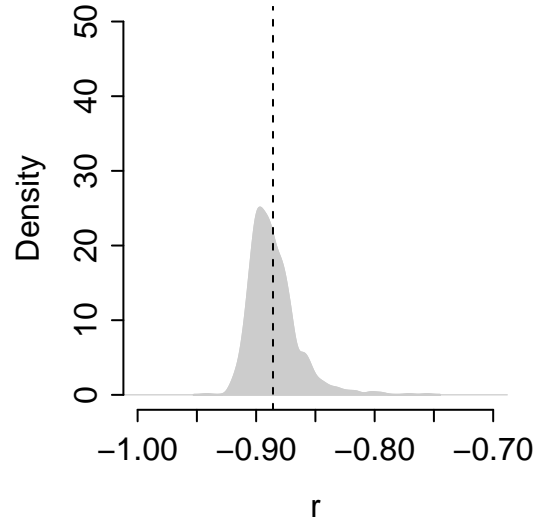*D. melanogaster*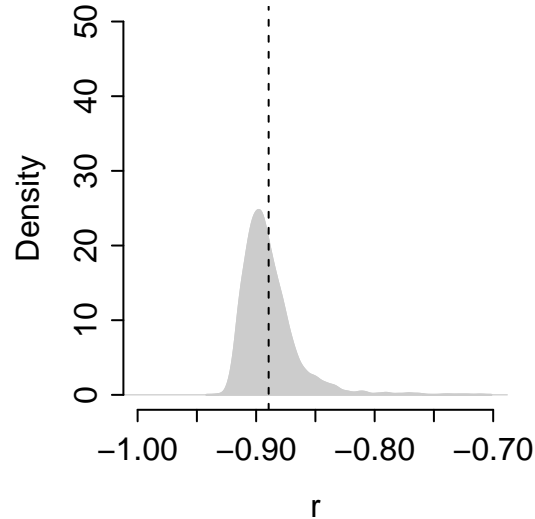*M. musculus*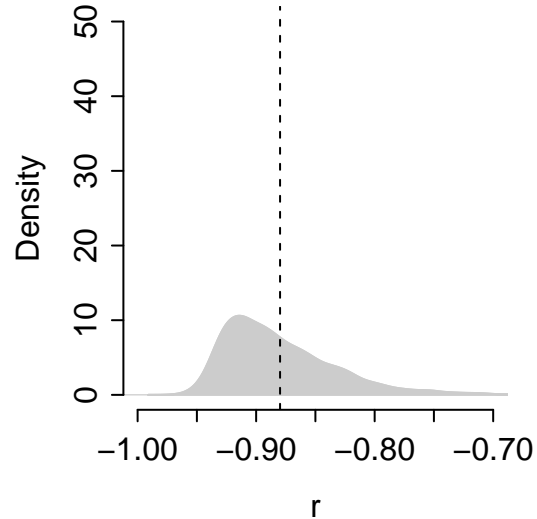

Supplement: Additional file 1: Figure S1 — Distribution of Pearson correlation coefficient between phyloP score and weighted entropy. Pearson correlation test was conducted for each gene. The dash line indicates the mean of Pearson correlation coefficient. [file 1471-2148-14-87-S1.pdf]

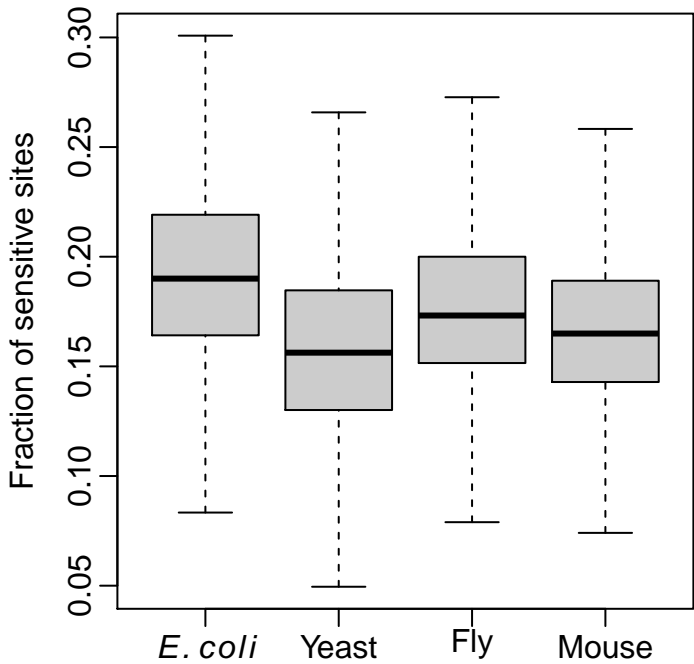

Supplement: Additional file 3: Figure S2 — Fraction of structurally sensitive sites in each species. We considered a nucleotide site as structurally sensitive if its structural sensitivity is larger than 0.1. [file 1471-2148-14-87-S3.pdf]

**A**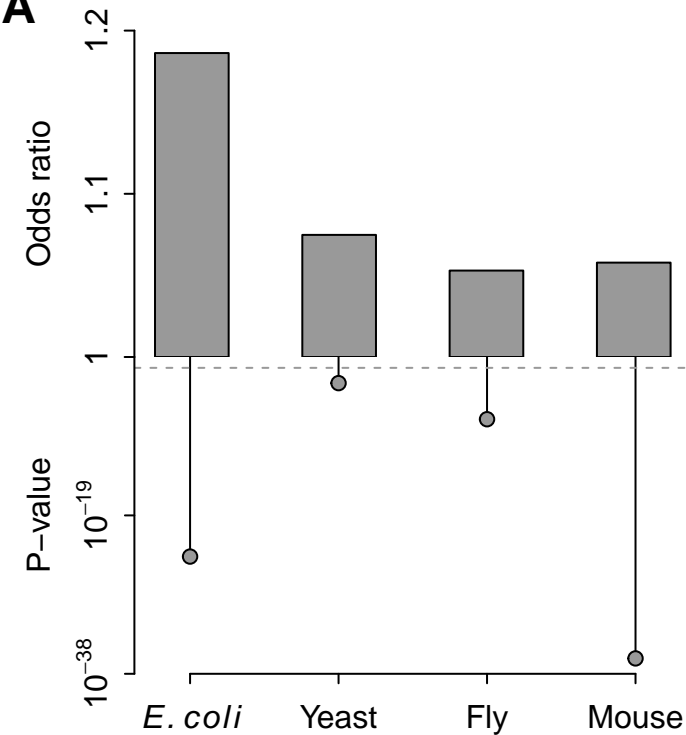**B**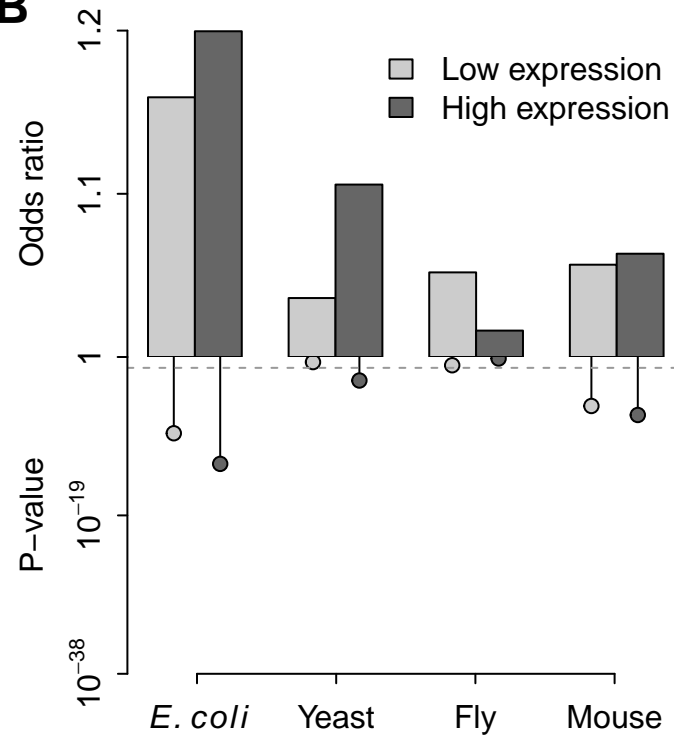**C**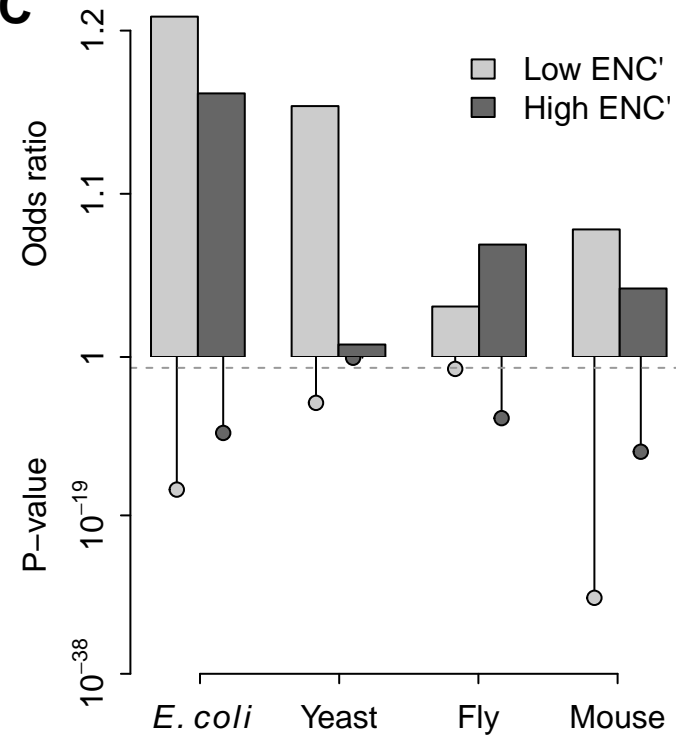

Supplement: Additional file 4: Figure S3 — Odds ratios and significance levels generated by Mantel-Haenszel procedure. We used phyloP conservation score as the measure of nucleotide conservation level. We considered sites with phyloP score > 0 as conserved. A) Comparison between species; B) Comparison between the 50% highest and lowest expressed genes; and C) Comparison between the genes with the top and bottom 50% ENC’ level. The dashed line denotes the significance level of α = 0.05. [file 1471-2148-14-87-S4.pdf]

*E. coli*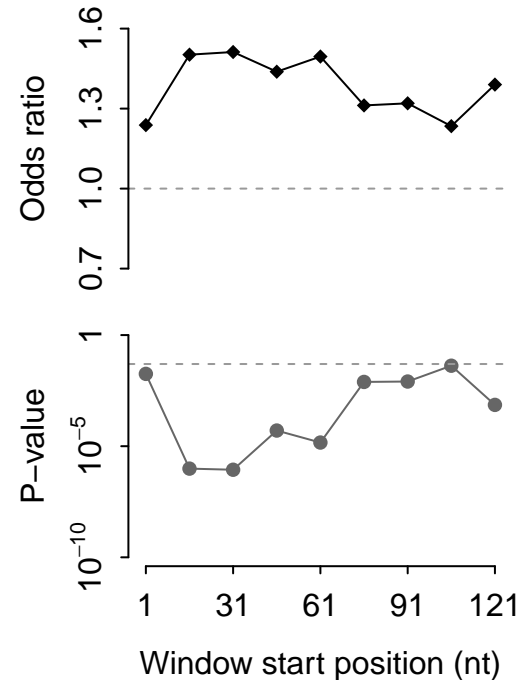*S. cerevisiae*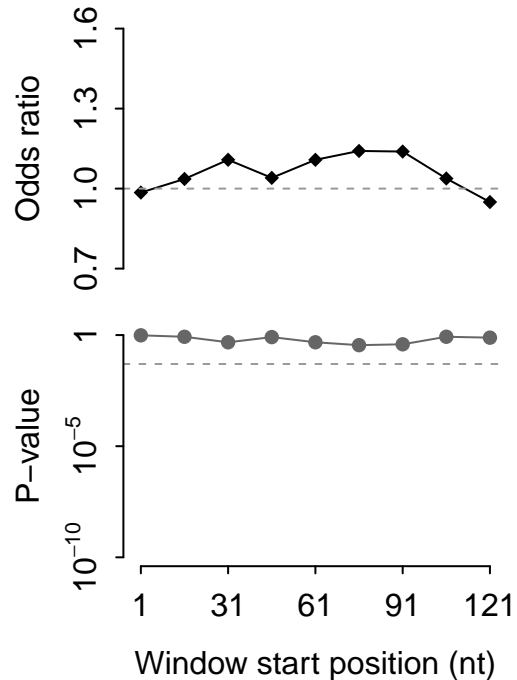*D. melanogaster*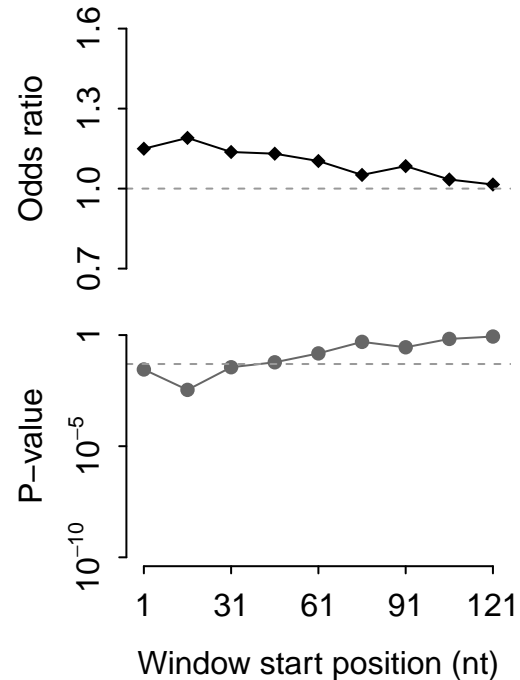*M. musculus*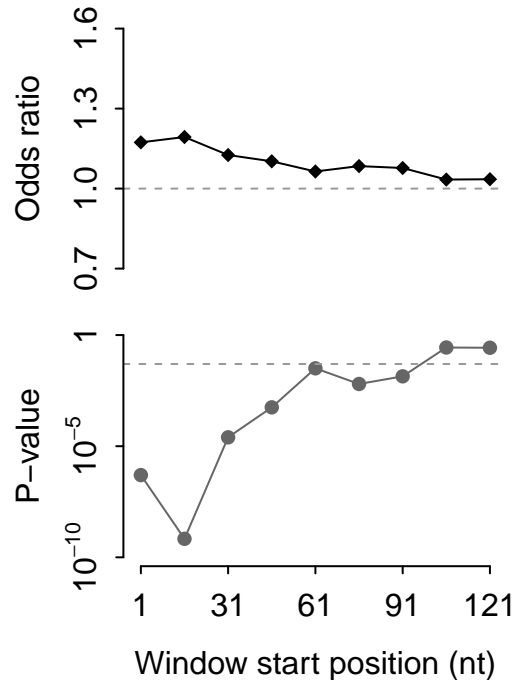

Supplement: Additional file 5: Figure S4 — The odds ratio and significance level of the 5′ sliding windows. We conducted Mantel-Haenszel test along the mRNA sequence using a sliding window of 45 nucleotides (nt) in length, moving from the start codon to the 121st downstream nucleotide in steps of 15 nt (for a total of 9 windows). The dashed lines in the lower panels denote the significance level of α = 0.05. [file 1471-2148-14-87-S5.pdf]

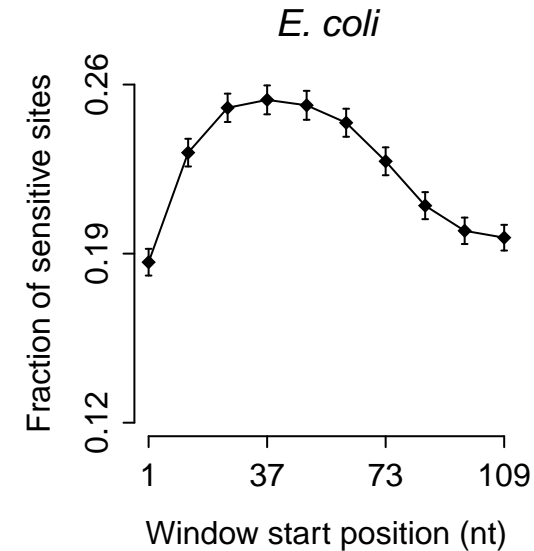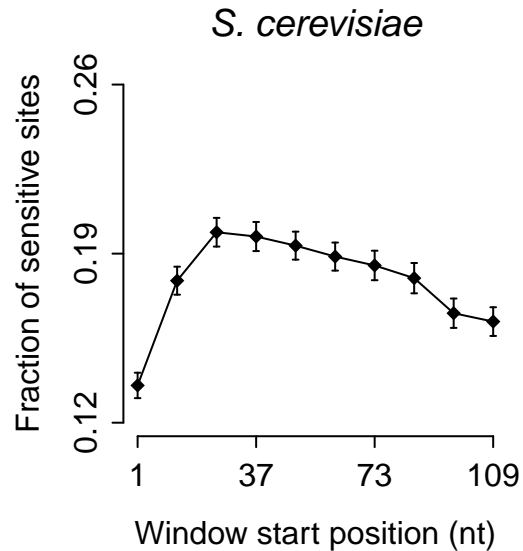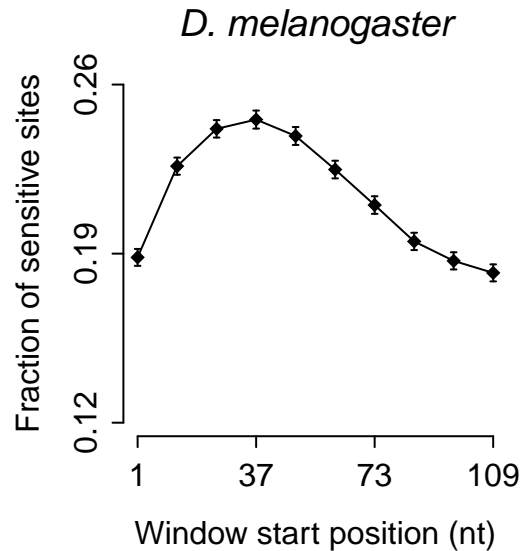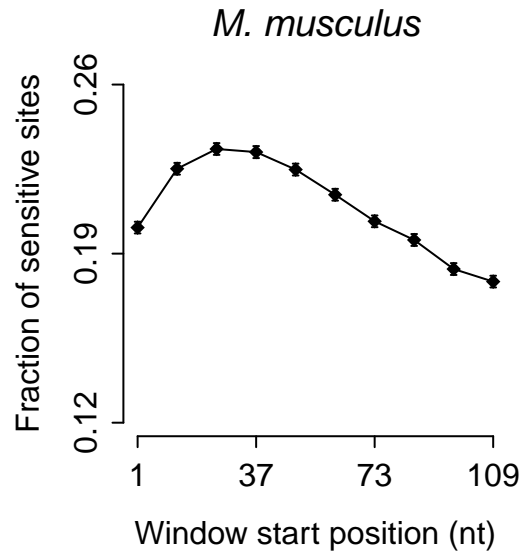

Supplement: Additional file 6: Figure S5 — Fraction of structurally sensitive sites of the 5′ sliding windows. We calculated the fraction of sensitive sites along the mRNA sequence using a sliding window of 36 nucleotides (nt) in length, moving from the start codon to the 109th downstream nucleotide in steps of 12 nt (for a total of 10 windows). [file 1471-2148-14-87-S6.pdf]

## Bacteria

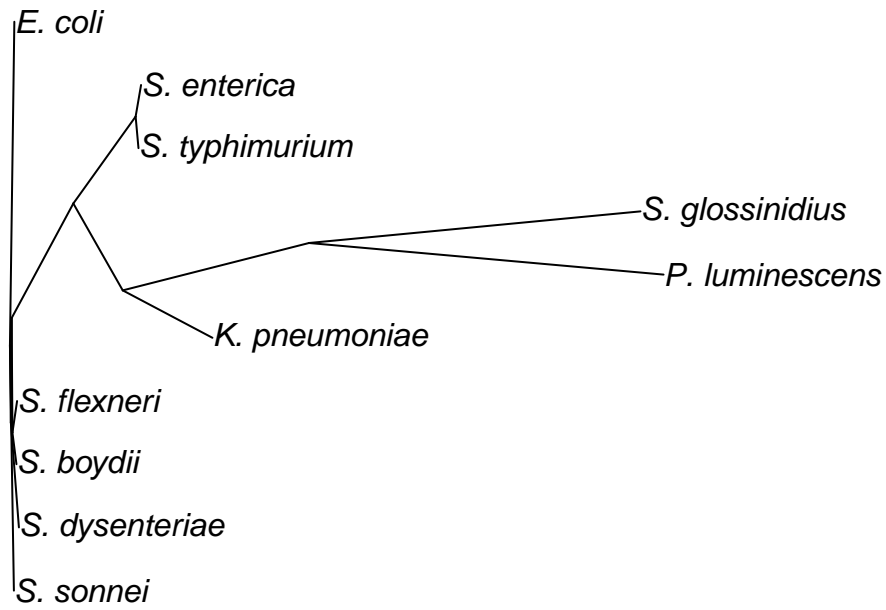

## Fungi

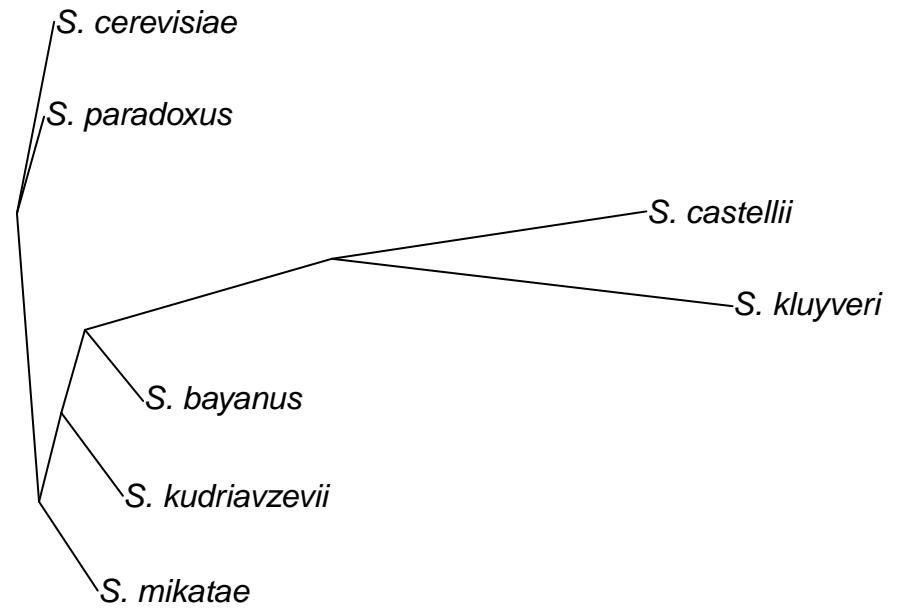

## Flies

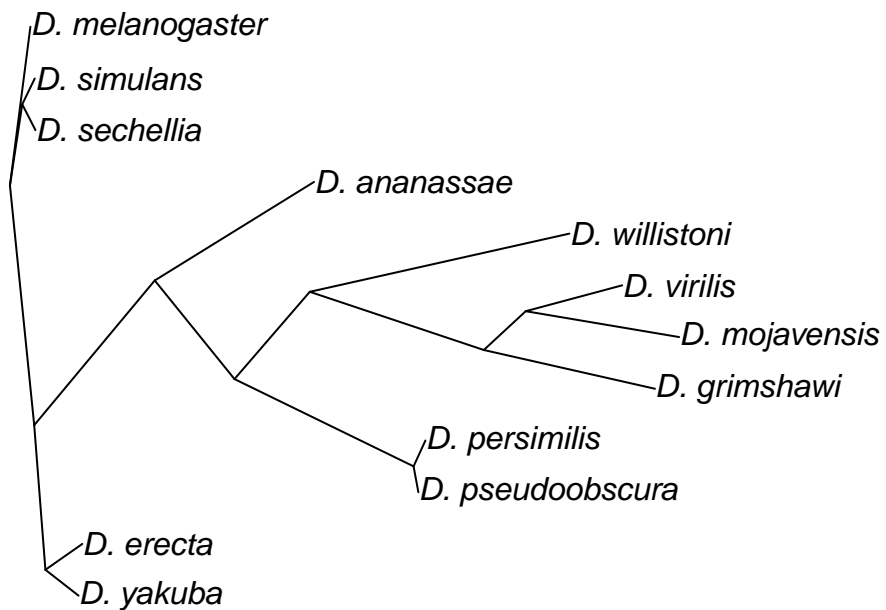

## Mammals

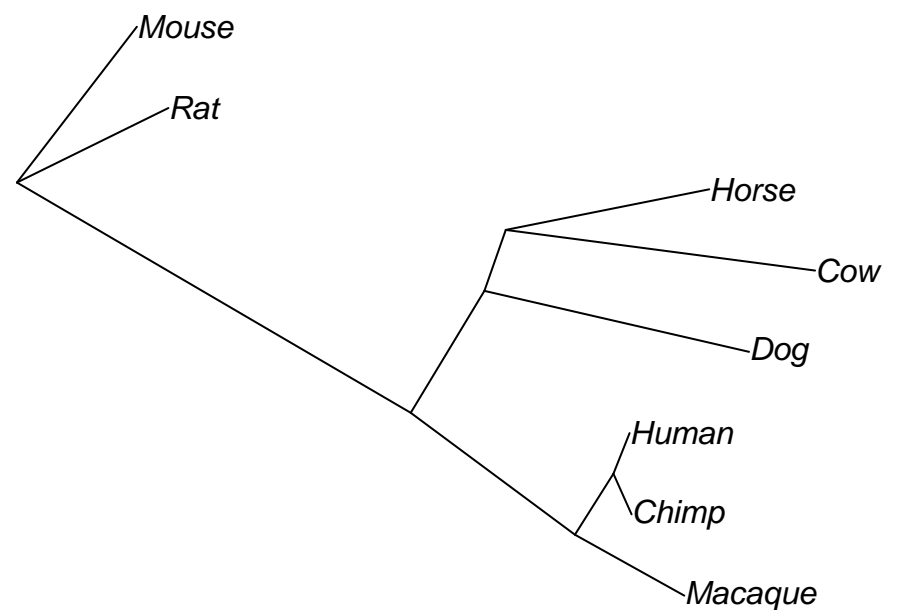

Supplement: Additional file 7: Figure S6 — Phylogenetic tree inferred by RAxML. Each phylogeny was estimated using the PROTGAMMABLOSUM62 model in RAxML. [file 1471-2148-14-87-S7.pdf]

*E. coli*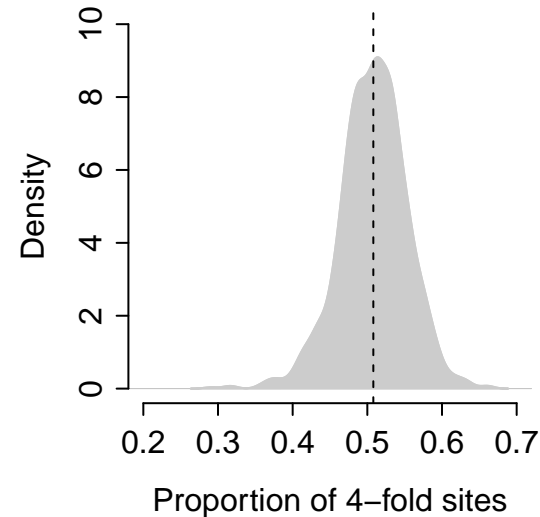*S. cerevisiae*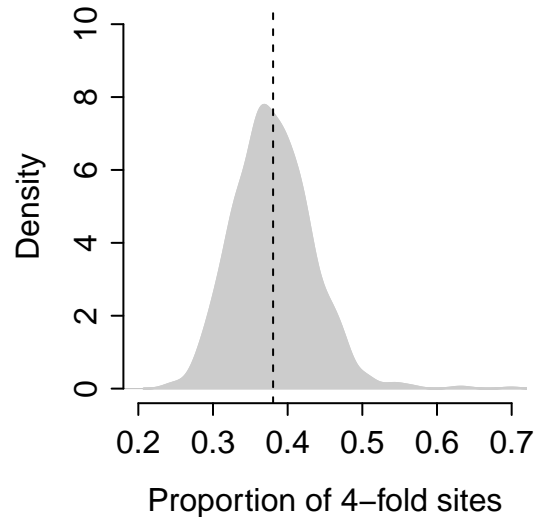*D. melanogaster*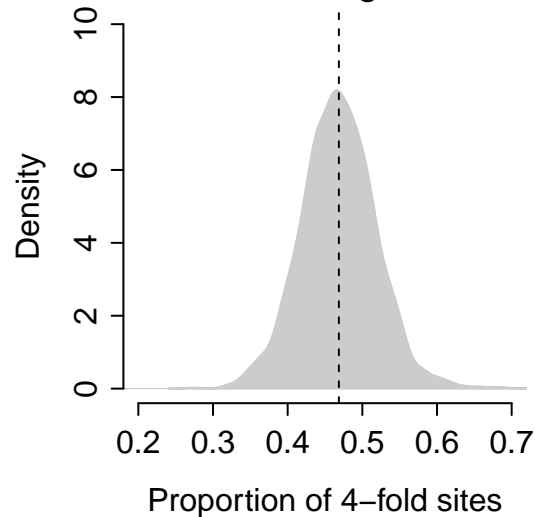*M. musculus*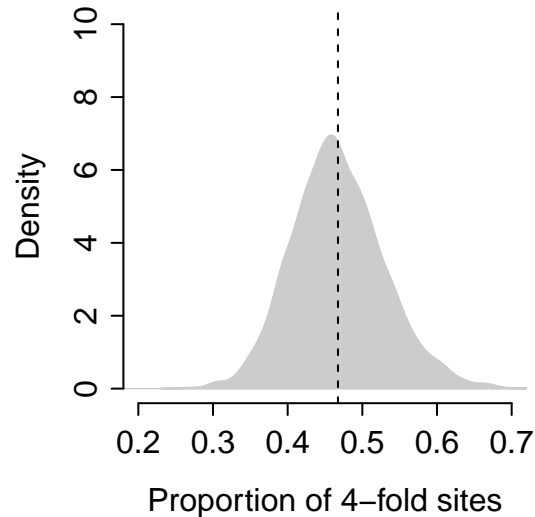

Supplement: Additional file 9: Figure S7 — Distribution of the proportion of 4-fold degenerate sites among the third codon positions in each gene. [file 1471-2148-14-87-S9.pdf]
