# Supplementary material for: The impact of RNA structure on coding sequence evolution in both bacteria and eukaryotes
Source: BMC Evol Biol. 2014 Apr 23;14:87. doi: 10.1186/1471-2148-14-87 (PMC4021280; doi:10.1186/1471-2148-14-87)
Supplement: Additional file 2: Table S1 — Odds ratio of conservation pattern between structurally disruptive and non-disruptive sites using different cutoffs. [file 1471-2148-14-87-S2.pdf]

Table S1. Odds ratio of conservation pattern between structurally disruptive and non-disruptive sites using different cutoffs

Cutoff of weighed entropy	Cutoff of structural sensitivity	E. coli		Yeast		Fly		Mouse	
		OR[a]	*P*	*OR*[a]	*P*	*OR*[a]	*P*	*OR*[a]	*P*
0.25	0.05	1.15	1.7E-18	1.03	1.8E-01	1.07	6.0E-11	1.02	7.2E-07
0.25	0.1	1.21	1.9E-23	1.07	3.5E-02	1.09	2.3E-10	1.04	2.2E-14
0.5	0.05	1.14	1.1E-17	1.04	3.7E-02	1.05	4.0E-09	1.02	8.6E-09
0.5	0.1	1.20	1.6E-21	1.07	1.5E-02	1.06	1.2E-07	1.04	2.3E-16

[a] *OR* is the abbreviation of odds ratio.
