# Supplementary material for: The impact of RNA structure on coding sequence evolution in both bacteria and eukaryotes
Source: BMC Evol Biol. 2014 Apr 23;14:87. doi: 10.1186/1471-2148-14-87 (PMC4021280; doi:10.1186/1471-2148-14-87)
Supplement: Additional file 8: Table S2 — Codons with 4-fold degenerate sites. [file 1471-2148-14-87-S8.pdf]

Table S2. Codons with 4-fold degenerate sites

Amino acid	Codons
Ala/A	GCU, GCC, GCA, GCG
Arg/R	CGU, CGC, CGA, CGG
Gly/G	GGU, GGC, GGA, GGG
Leu/L	CUU, CUC, CUA, CUG
Pro/P	CCU, CCC, CCA, CCG
Ser/S	UCU, UCC, UCA, UCG
Thr/T	ACU, ACC, ACA, ACG
Val/V	GUU, GUC, GUA, GUG
